# Pragmatic evaluation of prescription glucosamine sulfate (pGS) in knee osteoarthritis: insights from a Filipino cohort

**DOI:** 10.3389/fmed.2026.1878369

**Published:** 2026-07-20

**Authors:** Julie Yu, Juan Javier Lichauco, Sandra Navarra, Raffaella Maria Rita Chiaese, Srinivasan Venugopal, Scott Haughie, Chris Walker

**Affiliations:** 1 Department of Medicine, University of Santo Tomas Hospital, Manila, Philippines; 2 St. Luke's Medical Center, Manila, Philippines; 3 Global Medical Affairs, Viatris, Milan, Italy; 4 Biometrics, Viatris, Hyderabad, India; 5 Viatris/Mylan Pharma UK Ltd., Sandwich, Kent, United Kingdom; 6 Global Medical Affairs, Viatris, Hatfield, United Kingdom

**Keywords:** Filipino population, knee osteoarthritis (KOA), pain reduction, prescription glucosamine sulfate (pGS), real-world study, Symptomatic Slow-Acting Drugs for Osteoarthritis (SYSADOAs), WOMAC index

## Abstract

**Introduction:**

The real-world effectiveness and tolerability of prescription glucosamine sulfate (pGS) in reducing knee osteoarthritis (KOA) symptoms in a Filipino cohort was evaluated.

**Methods:**

This 8-week, pragmatic, open-label, multicenter study enrolled 281 Filipino adults with mild-to-moderate (Kellgren–Lawrence grade 2–3) KOA. Participants received pGS 1,500 mg once daily. Outcomes included changes from baseline assessed with the Western Ontario and McMaster Universities Osteoarthritis Index (WOMAC) and Visual Analog Scale (VAS) for pain at Weeks 4, 6, and 8. Tolerability was evaluated through adverse event monitoring.

**Results:**

Among 281 treated patients, 277 (98.6%) completed the study, and 245 (~88.0%) received pGS alone for the full period. These patients demonstrated rapid, sustained symptom improvement, with knee pain decreasing by Week 4 and continuing through Week 8. After 4 weeks, 132 patients (~47.0%) had already achieved ≥50.0% pain reduction. By Week 8, WOMAC pain showed a least squares (LS) mean change of −11.96 (95% CI: −12.26 to −11.67), with marked improvements in stiffness, physical function, and total score. VAS pain decreased from 68.4 mm at baseline to 11.9 mm at Week 8, consistent with meaningful clinical benefit. Treatment satisfaction was high, with 98.78% satisfied at Week 8 and a mean psychometric VAS score of 9.0. Patients with more advanced KOA requiring add-on therapy (*n* = 34) also demonstrated pain improvement, with an LS mean change in WOMAC pain of −8.81 (95% CI: −9.62 to −7.99). Overall, adverse events occurred in 4.6% of patients, were mostly mild, and led to only one withdrawal, confirming good tolerability of pGS as monotherapy and in multimodal treatment.

**Conclusion:**

Prescription glucosamine sulfate improved pain, stiffness, and function in Filipino patients with mild-to-moderate KOA as early as Week 4, sustained through Week 8. Treatment was well tolerated, with high satisfaction and one discontinuation. Most patients (~88%) were effectively managed with pGS monotherapy, while add-on celecoxib benefited those with insufficient response, supporting a multimodal approach for KOA symptom control in routine care.

## Introduction

Knee osteoarthritis (KOA) is the most common form of arthritis, characterized by intermittent weight-bearing pain that progresses to more persistent pain, stiffness, and physical disability (1, 2). Osteoarthritis (OA) symptoms impact quality of life commonly affecting daily activities and leading to deteriorating biopsychosocial function (3). Global estimates from 2020 highlighted that more than 590 million people were living with OA and, if the trend persists, it is estimated that nearly 1 billion subjects will experience some form of OA in 2050 (4). In the Philippines, in 2003, OA prevalence among individuals 40 years old and above was 0.5% (4). Many factors contribute to OA onset and progression. Older age is the greatest risk factor for OA (5). In 2020 OA was the seventh leading cause of years lived with disabilities (YLDs) for patients older than 70 and age standardized rate of YLDs were shown to be continuously increasing over time (6). Additionally, Western Pacific was one of the regions that has observed the greatest increase in life expectancy in the last 60 years (1950–2019) with an estimate of life expectancy reaching about 70 years old in the Philippines. Previous joint injury or overuse, gender (women are more affected than men) and being overweight/obese are also contributors for development of the condition (5). Obesity is often characterized by metabolic disorders and places an excessive load on the lower limb joints (6). The Western Pacific Region experienced one of the highest increases in adult obesity prevalence, from 2.7% in the 1970s to 6.4% in 2016 (7). At present, there is no known cure for OA and there are no licensed disease-modifying interventions. However, prescription glucosamine sulfate is a drug, obtained by a semi-synthetic route, which provides higher bioavailability and plasma concentrations in humans, leading to demonstrated clinical efficacy (8–13). In 2019, ESCEO (European Society for Clinical and Economic aspects of Osteoporosis, Osteoarthritis and Musculoskeletal Diseases) provided updated guidance (2) on knee OA treatment, suggesting the use of prescription glucosamine sulfate (pGS) or prescription grade chondroitin sulfate as symptomatic slow acting drugs for osteoarthritis (SYSADOAs) for background therapy and oral non-steroidal anti-inflammatory drugs (NSAIDs), administered subsequently or concomitantly, to acutely manage KOA related symptoms. In this pragmatic study, we described a large cohort of Filipino patients affected by KOA and their treatment with pGS over the 8-week study period, with the aim to measure the beneficial effect in real clinical practice in relieving KOA related symptoms.

## Patients and methods

### Study design

**Figure 1:**
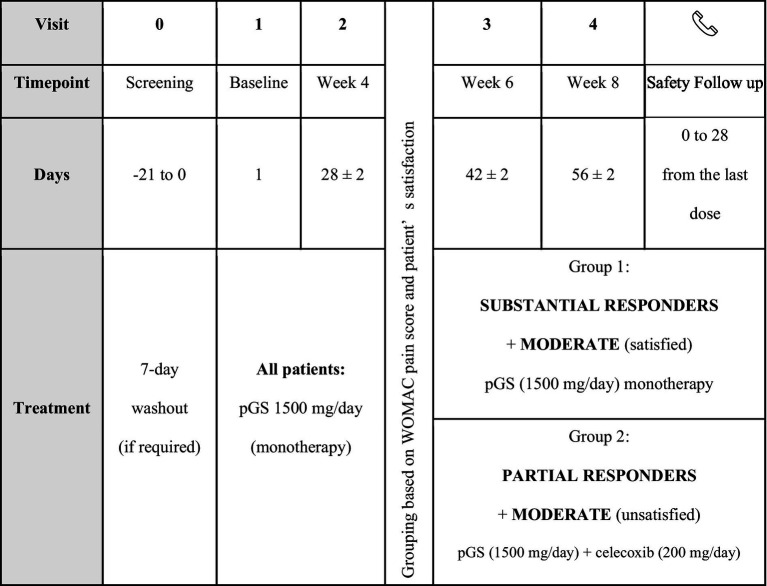
Study design overview. pGS, prescription glucosamine sulfate. Substantial responders: participants whose pain relief ≥50% vs. baseline; Moderate responders: participants whose pain relief ≥30–<50% vs. baseline; partial responders: participants whose pain relief <30% vs. baseline. Satisfaction: patient-reported VAS psychometric response (satisfied ≥5; unsatisfied <5).

This was an 8-week, pragmatic, parallel group, open-label, multicenter study conducted in three hospitals in Manila, Philippines between November 2022 and October 2024. The study protocol was approved by the Philippine Food and Drug Administration, by the local Institutional Review Board, and registered on the Philippines Clinical Trial Registry (2022-CT0712). The study was conducted in conformity with International Council for Harmonisation—Good Clinical Practice (ICH-GCP E6) guidelines, the Declaration of Helsinki, and the local regulatory requirements. All participants provided written informed consent prior to enrollment. The primary goal of the study was to evaluate the effectiveness of pGS in reducing KOA pain in a real-world Filipino cohort and to investigate the proportion of participants achieving clinically meaningful pain reduction (≥50%) or requiring an add-on therapy for advanced symptom control. The trial design is outlined in Figure 1.

### Study population

Eligible participants were adults between 50 and 70 years of age, with body mass index (BMI) < 29.99, a diagnosis of mild–moderate KOA, determined by the American College of Rheumatology clinical and radiological criteria (14) and/or grade 2 or 3 according to the Kellgren–Lawrence (KL) radiographic criteria (15, 16). A clear diagnosis of the OA severity was provided by the study investigators if a KL classification was missing in the X-Ray medical report. Patients at baseline (Visit 1) had a Western Ontario and McMaster Universities Osteoarthritis Index (WOMAC) score 40 and were either naive to pharmacologic treatments for OA or, if previously treated with oral or topical NSAIDs and/or analgesics, underwent a 1-week washout period prior to study entry. Patients were excluded if they had received any oral Symptomatic Slow-Acting Drugs for Osteoarthritis (SYSADOAs) or intra-articular (IA) injections with hyaluronic acid in the last 6 months, or IA injection of corticosteroids in the previous 3 months. For a full description of inclusion/exclusion criteria, please see Supplementary Table 1.

### Study treatments

All study participants received 8-week treatment with pGS (Viartril-S®, Rottapharm Ltd.) 1,500 mg powder for oral solution, approved as a once-daily dosage prescription medication for the treatment of KOA. After the initial 4 weeks, patients were assigned to receive either 1,500 mg/day of pGS monotherapy for an additional 4 weeks (Group 1) or receive 1,500 mg/day pGS with a COX-2 selective oral NSAID (celecoxib 200 mg/day, Celebrex®, Pfizer Pharmaceuticals LLC), based on the extent of their OA pain reduction and satisfaction score. More precisely, at Week 4, participants with substantial pain relief (≥50% reduction) maintained monotherapy treatment with pGS for a further 4 weeks (Group 1). Participants with partial pain relief (<30%) received celecoxib as per multimodal treatment strategy (Group 2) (2, 14). Those participants with moderate pain reduction (≥30 - < 50%), and based on their level of treatment satisfaction, had the option to maintain only pGS treatment, or add additional analgesia (celecoxib), based on the psychometric VAS response. Figure 1 shows patients grouping according to pain reduction threshold achieved.

Paracetamol (up to 2 g/day) was allowed as rescue medication exclusively in Group 1 (pGS monotherapy), with patient-reported use documented at each visit. Its use was prohibited during the 7 days prior to all efficacy assessments, thereby minimizing the likelihood that concomitant use of paracetamol contributed to the observed efficacy of pGS. The use of any other OA medication, i.e., other oral/topical NSAIDs, oral or injectable corticosteroids, and hyaluronic acid, was prohibited during the trial. Study drug adherence was assessed by counting all unused sachets and capsules that were brought back at each study visit.

### Assessments

Efficacy assessments were conducted at baseline and at Weeks 4, 6, and 8, according to the study visit schedule (Figure 1). Efficacy assessment was made using the standardized WOMAC index available in English language and in the 3.1 Tagalog Version.^1^ For a full description of WOMAC index, please see Supplementary Table 1.

The primary efficacy endpoint was to determine the severity of overall knee pain, from baseline to the end of treatment (8 weeks) by using WOMAC Total Pain sub-score (W-TPS). Secondary endpoint was to determine the severity of overall knee pain at 4 and 6 weeks using the same W-TPS. Further assessments, as secondary endpoints, were the change from baseline to 4, 6, and 8 weeks for the WOMAC Total score, the WOMAC Total stiffness sub-score (W-TSS), and the WOMAC Total Physical Function sub-scores (W-TPFS). Pain reduction was also assessed from baseline through 4, 6, and 8 weeks with the support of a 0–100 mm VAS (0 mm = no pain/none, 100 mm = severe intolerable pain). To determine the patient’s treatment satisfaction at Week 4 and 8, a 0–10 cm VAS psychometric response scale assessment was used (0 cm = extremely unsatisfied, 10 cm = extremely satisfied). The VAS psychometric response scale, which measures subjective characteristics or attitudes, has been used for a multitude of disorders, as well as in market research and social science investigations. Patients providing a rating ≥5 were deemed to be satisfied. Safety assessments, including physical examination, vital sign measurement, and routine safety laboratory tests were performed at screening and during study visits 2, 3, and 4.

### Statistical analyses

The sample size was determined, assuming that approximately 60% of participants will require additional therapy from week 4, and the remaining 40% will continue monotherapy. Simulated data from WOMAC pain results from literature (13, 17, 18) suggests the mean change W-TPS should be −8.35 (SD 2.52) from baseline. Assuming that 150 participants in the dual therapy group would provide a precision of 0.403 [half width of 95% confidence interval (CI)] for the mean change W-TPS at Week 8 and adjusting 10% of participants not available for evaluation, 167 participants were expected to receive dual therapy while 113 to receive monotherapy. Hence, a total sample size of 280 patients was enrolled.

Changes from baseline for W-TPS at Week 8, were analyzed using an analysis of covariance (ANCOVA) model, including treatment as a factor and baseline W-TPS score as a covariate. The LS mean changes and corresponding 95% CI at Week 8 were estimated for each treatment group. Similarly, for WOMAC Total, W-TPFS, W-TSS sub-scores, and VAS pain, the 95% CI for the mean difference and *p*-value were calculated using a paired *t*-test. To further assess the robustness of the primary analysis and address potential residual confounding due to baseline differences between treatment groups, a sensitivity analysis was performed using an extended ANCOVA model. In this model, additional baseline covariates were included, specifically KL radiographic grade (Grade 2 vs. Grade 3) and joint involvement status, alongside baseline WOMAC pain score and treatment group. As radiographic KL grading was available only for a subset of participants at baseline (*n* = 79 out of 281), inclusion of this variable as a standard categorical covariate in a complete-case analysis would have resulted in a substantial reduction in sample size. Therefore, to preserve the integrity and size of the Full Analysis Set (FAS), participants without available KL grading data were retained by assigning a dedicated “Not Done” category for this variable. The sensitivity ANCOVA model therefore included treatment as a factor, baseline WOMAC pain score as a covariate, and KL grade and joint involvement status as additional categorical covariates. LS mean changes from baseline and corresponding 95% CI were estimated for each treatment group.

All efficacy analyses were based on the FAS defined as all enrolled patients who were confirmed to have knee OA, received study treatment, and had an evaluable assessment for the primary efficacy endpoint (baseline W-TPS and at least one post-baseline value). The Safety Analysis Set (SS) included all participants who received at least one dose of any study treatment. Adverse events were summarized descriptively by Preferred Term (PT) and System Organ Class (SOC) using the Medical Dictionary for Regulatory Authorities (MedDRA, most recent version).

## Results

**Figure 2:**
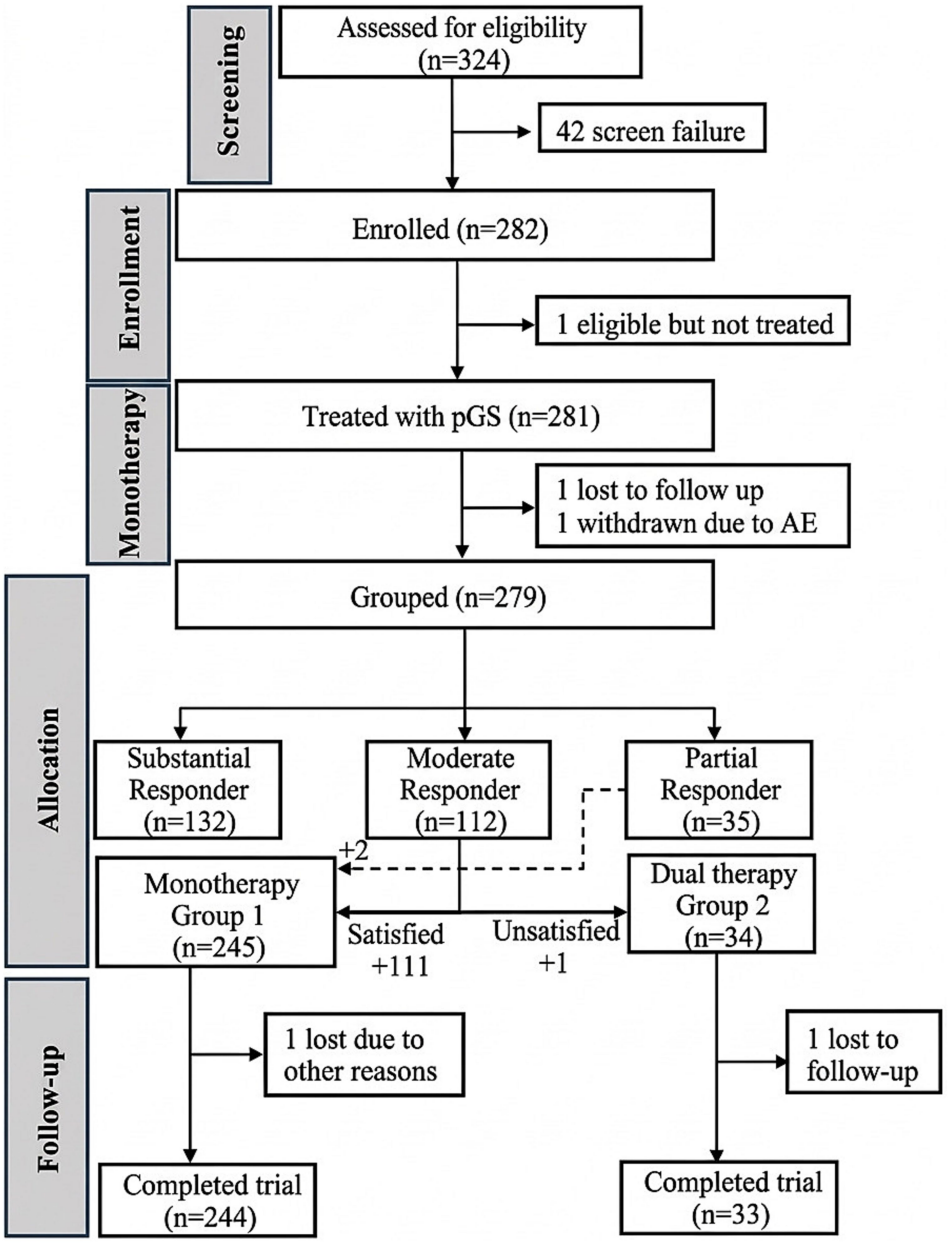
Patient flow diagram (safety set). pGS, prescription glucosamine sulfate; AE, adverse events; dotted line indicates the two partial responders who were erroneously assigned to Group 1.

A total of 324 participants were screened; 281 patients were treated and 277 (98.6%) completed the study. Patient disposition is summarized in Figure 2, and baseline demographic and clinical characteristics are reported in Table 1. Notably, of all the treated patients, 83.3% were female and 87.9% were diagnosed with OA for less than 5 years before the date of first OA treatment. FAS included 279 (99.3%) of the treated participants and SS included 281 (100%).

**Table 1:** Baseline characteristics of patients in SS (*N* = 281) population.

Characteristics	Total (*N* = 281)	Group 1 (*N* = 245)	Group 2 (*N* = 34)
Age, years			
Mean ± SD (range)	59.3 ± 5.21 (50–69)	59.3 ± 5.20 (50–69)	59.9 ± 5.33 (50–69)
Gender			
Male, *N* (%)	47 (16.7)	43 (17.6)	4 (11.8)
Female, *N* (%)	234 (83.3)	202 (82.4)	30 (88.2)
Height, cm			
Mean ± SD (range)	155 ± 9.0 (128–193.5)	154.9 ± 9.03 (128–193.5)	155.5 ± 9.05 (138–185)
Weight, kg			
Mean ± SD (range)	59.5 ± 9.47 (33.4–83.5)	59.3 ± 9.66 (33.4–83.5)	60.3 ± 8.14 (47–81)
BMI, kg/m^2^			
Mean ± SD (range)	24.8 ± 3.51 (15.2–29.9)	24.7 ± 3.61 (15.2–29.9)	25.0 ± 2.86 (18.1–29.8)
OA KL grading^a^			
Grade 2, *N* (%)	15 (5.3)	11 (4.5)	4 (11.8)
Grade 3, *N* (%)	64 (22.8)	45 (18.4)	18 (52.9)
OA severity by PI^b^			
Mild, *N* (%)	148 (52.7)	141 (57.6)	6 (17.6)
Moderate, *N* (%)	54 (19.2)	48 (19.6%)	6 (17.6)
Co-morbidities			
Dyslipidaemia, *N* (%)	26 (9.3%)	19 (7.8)	7 (20.6)
Hypertension, *N* (%)	114 (40.6)	98 (40.0)	16 (47.1)
Hypothyroidism, *N* (%)	4 (1.4)	2 (0.8)	2 (5.9)

SS, safety set; BMI, Body Mass Index; OA, osteoarthritis; KL, Kellgren Lawrence; PI, principal investigator.

a Severity of OA according to radiographic diagnosis when KL classification was included in documentary evidence of an X-ray performed within the previous year.

b Severity of OA according to Principal Investigator’s discretion when X-Ray was performed at Site Visit (screening).

Following the Week 4 assessment, most participants continued pGS monotherapy (*n* = 245), whereas a smaller subgroup (*n* = 34) with insufficient pain reduction (<30%) or lower satisfaction (psycho VAS < 5) required treatment intensification with add-on therapy (Group 2). Minor protocol deviations in group allocation were identified, with two partial responders incorrectly assigned to Group 1 due to a miscalculation; however, this had a negligible impact given the small number involved.

The sample size assumption that 40% of patients would remain on pGS monotherapy until Week 8 proved to be inaccurate, as a greater percentage of patients (~88%) were successfully managed with monotherapy. Only a small number of patients required additional analgesia to achieve at least 50% pain reduction, reflecting the inclusion of a population with a relatively mild disease phenotype, for whom single-agent therapy is often sufficient, in line with established treatment guidelines and algorithms. A flow diagram of patients is shown in Figure 2, and baseline demographic data are shown in Table 1.

At the end of 8 weeks, Group 1 had an improvement in W-TPS, with a mean change from baseline (CFB) of −12.0 (95% CI; −12.40, −11.62; *p* < 0.0001). This reduction was already clinically relevant at Week 4, where the change from baseline in W-TPS was −9.8 (95% CI; −10.15, −9.50; *p* < 0.0001) and this was sustained at Week 6 with a change of −11.00 (95% CI; −11.44, −10.66; p < 0.0001). Similarly, the mean CFB in W-TOTAL, was clinically relevant (*p* < 0.001) across the study: -45.7 (95% CI; −47.38, −44.03), −54.7 (95% CI; −56.43, −52.93) and −58.9 (95% CI; −60.55, −57.16) respectively at Week 4, 6 and 8. Observed mean (SD) scores for VAS pain and are provided in Table 2 across the different study timepoints.

**Table 2:** Efficacy outcomes in patients with knee OA receiving pGS (1,500 mg/daily) in monotherapy for 8 weeks in the FAS (*N* = 279) population.

Measure	Baseline	Week 4	Week 6	Week 8
VAS pain mean ±SD	68.4 ± 10.31	25.2 ± 12.15	19.2 ± 13.23	11.9 ± 12.33
W-TPS mean ± SD	13.5 ± 2.63	3.8 ± 2.20	2.6 ± 2.38	1.6 ± 2.10
W-TSS mean ± SD	5.6 ± 1.37	1.7 ± 1.32	0.9 ± 1.09	0.5 ± 0.90
W-TPFS mean ± SD	46.7 ± 8.47	14.8 ± 8.85	7.8 ± 7.71	4.9 ± 5.94
W-TOTAL mean ± SD	65.9 ± 11.46	20.02 ± 11.49	11.3 ± 10.68	7.0 ± 8.43

OA, osteoarthritis; pGS, prescription glucosamine sulfate; SD, standard deviation; FAS, full analysis set; VAS, visual analog scale of 0–100 mm; WOMAC, Western Ontario and McMaster Universities Osteoarthritis Index; W-TPS, WOMAC total pain sub-score; W-TSS, WOMAC total stiffness sub-score; W-TPFS, total physical function sub-score; W-TOTAL, WOMAC total sub-score.

The LS mean changes for W-TPS, W-TPFS, W-TSS and W-TOTAL at Week 8 were, respectively, −11.96 (95% CI; −12.26 to −11.67), −41.99 (95% CI; −42.89 to −41.09), −5.05 (95% CI; −5.18 to −4.93), and −59.00 (95% CI; −60.27, −57.74). Benefits of pGS were consistently reflected in the high level of patient treatment satisfaction, as evidenced by the Psychometric VAS scores. At Week 8, 242 of 245 (98.78%) participants receiving pGS (monotherapy) reported satisfaction, with a Mean (SD) Psychometric VAS score of 9.0 (1.22).

Patients in Group 2, characterized by moderate knee OA (70% of participants in this group were diagnosed with KL 3) affecting more than one joint (100% of participants), achieved an improvement at Week 8 (*p* < 0.0001) with a LS mean change in W-TPS of −8.81 (95% CI; −9.62, −7.99) from baseline. Specifically, between Weeks 4 and 8, Group 2 experienced a mean reduction of 5.7 points in W-TPS, whereas Group 1, which did not receive additional NSAID, demonstrated a minor reduction of 2.2 points during the same interval.

The exploratory efficacy results for Group 2, including secondary outcomes, are presented in Supplementary Table 2. Due to the limited number of patients in Group 2 and the inadequate sample size, analyses are limited, and these findings should be interpreted with caution in light of potential residual confounding.

To evaluate the impact of baseline structural differences between treatment groups, a sensitivity analysis was conducted using an extended ANCOVA model including additional covariates (KL grade and joint involvement status). The results of this adjusted model are presented in Table 3. The adjusted analysis demonstrated that the treatment effect remained consistent with the primary model. Specifically, the LS mean changes in WOMAC pain scores at Week 8 were −10.66 for Group 1 and −7.30 for Group 2 demonstrating that the magnitude of the difference between treatment groups was therefore preserved. These findings indicate that adjustment for baseline structural characteristics did not materially influence the observed treatment effect, supporting the robustness of the primary analysis.

**Table 3:** Analysis of covariance for mean change in severity of WOMAC sub scale for knee pain from baseline to week 8 in FAS (*N* = 279) population.

Visit/statistics	Treatment group	
	pGS monotherapy (*N* = 245)	pGS + celecoxib (*N* = 34)
Visit 4/Week 8		
*n*	244	32
LS mean^a^	−10.66	−7.30
Standard error	0.38	0.54
95% CI for LS mean	(−11.41, −9.90)	(−8.37, −6.23)

WOMAC, Western Ontario and McMaster Universities Osteoarthritis Index; FAS, full analysis set; pGS, prescription glucosamine sulfate; LS, least square; CI, confidence interval.

a The LS mean change in severity of WOMAC sub-scale score for knee pain from baseline to week 8 were calculated by using ANCOVA model where baseline WOMAC pain score as a covariate, KL classification grade, knee joint involved and Treatment as a factor and change in WOMAC score were dependent variable. Womac sub scale for knee pain data is available for only 276 subjects at Visit 4.

Overall, the study treatment was well tolerated in the real-life Filipino KOA cohort. Adverse events (AEs) were reported in a small proportion of patients (4.6%), were all non-serious and mostly mild. Treatment withdrawal was required only for 1 participant (0.4%) due to dyspepsia in association with pGS administration during the first 4 weeks, while temporary discontinuation was required for two participants. No clinically meaningful changes were observed in physical examination and vital signs, or laboratory parameters. A detailed summary of adverse events by treatment group and study period is provided in Supplementary Table 3.

## Discussion

Prescription glucosamine sulfate is one of the first-line drugs recommended by the ESCEO working group for treating mild to moderate KOA (2). Whilst the medicine belongs to a class of SYSADOA, pGS has shown symptom-modifying effect in medium- to long-term studies, with efficacy as early as Week 4 (12, 13, 18). Owing to its favorable pharmacokinetic profile, pGS (1,500 mg/day) achieves steady-state plasma concentrations of approximately 10 μM following oral administration, which are sufficient to exert pharmacological effects, including inhibition of NF-κB–mediated inflammatory pathways, reduction of cartilage degradation, and promotion of joint homeostasis (8, 19–21). The literature findings are consistent with the outcomes of this 8-week pragmatic study, suggesting that pGS is an effective and well-tolerated treatment for knee OA symptoms. The primary result of this study indicated that pain reduction was clinically relevant from Week 4, and the improvement continued steadily until Week 8 with a sustained relief from all OA symptoms in accordance with literature outcomes. Indeed, most patients (87%) experienced clinically relevant pain reduction (≥50%) by Week 4, in line with the relatively mild baseline disease status. The effects observed in this open label study are larger than those observed in randomized controlled trials (13, 17) where WOMAC was utilized to assess pain, stiffness and function. Open label consideration of pain medicines has traditionally demonstrated larger treatment effects for pain relief than observed in RCTs across different types of chronic pain (22–24). Work presented in the literature, characterizing the pain trajectories present in patients who were at high risk of developing or already had OA of the knee, has illustrated that the largest sub-cohort of patients had a mild non progressive phenotype (25). This population, all other factors considered, would be expected to be largely treated singularly by agents early in ESCEO’s suggested treatment algorithm (SYSADOA) (2). However, OA is a condition where different pain trajectories are present (25) and the disease may show progressive worsening or undergo periods of intermittent worsening. In either scenario a more aggressive strategy may be required. As previously suggested (26), combining glucosamine sulfate with NSAIDs represents a promising multimodal approach for managing KOA, with potential benefits on pain relief, functional improvement, and modulation of inflammatory and cartilage degradation processes. Interestingly, this cohort of Filipino patients was mainly characterized by relatively early stage OA (approximately 88% had the disease for <5 years), and mild, by physician-assessed disease (around 60% of participants), with patients with severe symptomatic OA excluded by design. Even if in the literature there is a limited relationship between radiographic findings and OA symptoms, the disease phenotype treated in this trial is largely consistent with SYSADOA only therapy, with additional analgesia through NSAID usage being utilized commonly in moderate to severe disease stages. Notably, although the present study focuses on KOA, emerging evidence (27, 28) suggests that pGS may also provide symptomatic benefits in other OA localizations such as hand. It is important to underscore the need to integrate symptom-based measures when evaluating treatment response, particularly in pragmatic, real-world settings to tailor patient treatment.

The trial has some limitations. The absence of a placebo group means that the true effect of the interventions at both Week 4 and Week 8 is partially attributable to a placebo effect. However, the study was designed to reflect a relatively naturalistic setting, with a 1-week washout to induce a flare state to encourage a more consistently measurable response to pharmacotherapeutic interventions. Group 2, requiring additional analgesia, was small in number, not based on a formal sample size calculation, and had substantially different baseline clinical characteristics compared with Group 1. Consequently, findings from the multimodal strategy (SYSADOA + NSAID) should be considered exploratory and offer only preliminary insights into the management of patients with an insufficient early response to monotherapy. Nevertheless, the observed outcomes are consistent with previous studies suggesting a potential synergistic effect between pGS and celecoxib (26, 29, 30). It is also possible the dominant grouping, at Week 4, for the moderate responders towards monotherapy was influenced by aspects of Filipino culture. The study enrollment was largely completed thanks to word of mouth in the community. Filipinos cope with ill health through support from family, friends, and religion. They trust clinicians with positive tone and clear communication (31). The slightest pain reduction or physical improvements may have been perceived as highly positive, producing a positive (≥5) rating when the Psychometric VAS for treatment satisfaction was assessed. As mentioned by Roditi and Robinson (32), a pattern identified in chronic pain research is “the possibility that the effects captured are primarily satisfaction with treatment delivery rather than actual satisfaction with treatment effects”. Future trials studying patient preference, should consider this distinction to effectively probe the determinants of patient satisfaction more effectively. Nevertheless, providing adequate pain relief, as early as possible in the disease life cycle, is an important step to maintaining patients’ intrinsic capacity, and avoiding long-term disability (33). Current evidence indicates that achieving a ≥50% decrease in pain is a clinically meaningful target for OA treatment, and it is considered a substantial improvement that promotes functional preservation and contributes to favorable long-term outcomes (34). The results of this real-life, pragmatic study on the reduction of KOA symptoms in an Asian population are the first to suggest that pGS is an effective background drug for achieving clinically relevant pain reduction and physical function improvement for KOA management.

## Data Availability

The raw data supporting the conclusions of this article will be made available by the authors, without undue reservation.

## Footnotes

1
https://www.womac.com/womac/
