# Supplementary material for: Pragmatic evaluation of prescription glucosamine sulfate (pGS) in knee osteoarthritis: insights from a Filipino cohort
Source: Front Med (Lausanne). 2026 Jul 20;13:1878369. doi: 10.3389/fmed.2026.1878369 (PMC13431446; doi:10.3389/fmed.2026.1878369)
Supplement: Supplementary file 1 [file Table_1.docx]

***Supplementary Material***

**Supplementary Materials - Table 1:** Study Protocol details

| **PRIMARY OBJECTIVE** | - To evaluate the change in the severity of overall knee pain from baseline to end of treatment (at 8 weeks) as assessed using the Western Ontario and McMaster Universities Arthritis Index (WOMAC pain subscale score).   WOMAC is a self-assessed disease-specific measure for subjects with OA of the knee and hip, comprising 24 items in three dimensions: pain, function, and stiffness. Each item is scored on 4 grades: 0 point (none), 1 point (slight), 2 points (moderate), 3 points (very), and 4 points (extremely), and the total score is 96 points. There are 5 items related to pain (total score of 20 points), 2 items to joint stiffness (total score of 8 points), and 17 items to joint function (total score of 68 points). A higher score corresponds to more severe KOA symptoms in subjects.   - Pain (5 items): during walking, using stairs, in bed, sitting or lying, and standing upright - Stiffness (2 items): after first waking and later in the day - Physical Function (17 items): using stairs, rising from sitting, standing, bending, walking, getting in / out of a car, shopping, putting on / taking off socks, rising from bed, lying in bed, getting in / out of bath, sitting, getting on / off toilet, heavy domestic duties, light domestic duties |
| --- | --- |
| **SECONDARY OBJECTIVE** | - To determine the severity of overall knee pain, overall knee function, overall knee stiffness and overall KOA symptoms from baseline through 4, 6, and 8 weeks of treatment by using WOMAC pain subscale; WOMAC function subscale, WOMAC stiffness subscale, TOTAL WOMAC score and Visual Analogue Scale (VAS) scale assessment.   VAS pain scale is the most validated and frequently evaluated 100-mm visual analogue scale for pain assessment, providing excellent reliability. (VAS pain intensity, 0 = no pain/none, 100 = severe intolerable pain).   - To assess the subject’s treatment preference and satisfaction at Week 4 (baseline) and Week 8 versus Week 4 using a 0-10 cm VAS (psychometric response) scale assessment.   The VAS (psychometric response) scale is used to measure subjective characteristics or attitudes and has been used in the past for a multitude of disorders, as well as in market research and social science investigations. VAS (psychometric response) 10-cm scale is used to rate the subject’s treatment satisfaction with 0 = extremely unsatisfied and 10 = extremely satisfied.   - To assess the safety of the study treatment |
| **EXCLUSION CRITERIA** | - Patients were excluded if known concomitant rheumatic diseases (e.g., rheumatoid arthritis, psoriatic arthritis) or a history or clinical evidence of other inflammatory diseases or current treatment with immunosuppressive agents or oral glucocorticoids. - Patients were excluded if they had active peptic ulcer or GI bleeding; history of asthma, acute rhinitis, nasal polyps, angioedema, urticaria or other allergic-type reactions to acetylsalicylic acid or other NSAIDs; if affected by cardiac, renal, or hepatic disease; or were taking oral coumarin anticoagulants, angiotensin receptor blockers (ARBs), angiotensin converting enzyme (ACE) inhibitors, or diuretics with eGFR <60 ml/min. - Female patients were excluded if pregnant or breast-feeding or in pre-menopause. |
| **INCLUSION CRITERIA** | - Male and female subjects between 50 and 70 years of age. - Female subjects: Postmenopausal (amenorrheic for ≥12 months, confirmed by menstrual history) or documented hysterectomy and/or bilateral oophorectomy. - Subjects matched ACR criteria for the diagnosis of knee osteoarthritis (OA) with radiographic diagnosis of KOA in at least one knee and with Kellgren-Lawrence (KL) grade 2–3. X-ray performed at screening or documented X-ray within past year. - If KL grading not used (e.g., Philippines), severity must be clearly stated (mild, moderate, severe). - Only mild to moderate KOA included. - WOMAC Total score at baseline (Visit V1) ≥ 40 - Subjects naive to pharmacologic treatments for OA or have completed a 1-week washout if previously on treatment with paracetamol and/or oral/topical NSAIDs - Subjects were not required to change their dietary regimen during the study. - Subjects were willing to comply with the requirements of the study protocol, including acceptance of regular blood sampling. - Written informed consent |

**Supplementary Materials - Table 2:** Efficacy outcomes in patients with knee OA receiving dual therapy (pGS 1500 mg/day + celecoxib 200 mg/day) from Week 4 up to Week 8 in the FAS (N=279) population.

| **Measure** | **Week 4** | **Week 6** | **Week 8** |
| --- | --- | --- | --- |
| **VAS Pain mean ± SD** | 49.1 (20.99) | 27.9 (17.04) | 25.5 (19.95) |
| **W-TPS mean ± SD** | 10.5 (3.38) | 5.3 (3.51) | 4.7 (3.98) |
| **W-TSS mean ± SD** | 4.6 (1.73) | 2.1 (1.55) | 1.9 (1.74) |
| **W-TPFS mean ± SD** | 36.7 (13.84) | 18.2 (12.37) | 15.8 (13.67) |
| **W-TOTAL mean ± SD** | 51.8 (18.23) | 25.6 (16.90) | 22.3 (19.16) |
| OA = osteoarthritis; pGS = prescription Glucosamine Sulfate; FAS = full analysis set; SD = standard deviation; VAS = visual analog scale of 0 to 100 mm; WOMAC = Western Ontario and McMaster Universities Osteoarthritis Index; W-TPS = WOMAC total pain sub-score; W-TSS = WOMAC total stiffness sub-score; W-TPFS = total physical function sub-score; W-TOTAL = WOMAC total sub-score. | | | |

**Supplementary Materials – Table 3**: Summary of Adverse Events in the Safety Set analysis (N=281).

|  | Treatment Group (Up to Week 4) | Treatment Group  (Week 4-8) | |
| --- | --- | --- | --- |
| **Side effects,**  n (%) | **pGS monotherapy**  **(n=281)** | **pGS monotherapy**  **(n=245)** | **pGS + celecoxib**  **(n=34)** |
| **Acid peptic disease** | **-** | **-** | 1 (2.9%) ** |
| **Diarrhea** | **-** | 1 (0.4%) | 1 (2.9%) |
| **Dyspepsia** | 1 (0.4%) * | **-** | 1 (2.9%) |
| **Gastroenteritis** | 1 (0.4%) ** | - | **-** |
| **Somnolence** | 1 (0.4%) | - | **-** |
| **Headache** | 1 (0.4%) | - | **-** |
| **Hypertension** | **-** | 4 (1.6%) | 2 (5.9%) |
| pGS, prescription glucosamine sulfate.  *Treatment withdrawn; **Temporary treatment discontinuation | | | |
